# Blueprints for Biosensors: Design, Limitations, and Applications

**DOI:** 10.3390/genes9080375

**Published:** 2018-07-26

**Authors:** Alexander C. Carpenter, Ian T. Paulsen, Thomas C. Williams

**Affiliations:** 1 Department of Molecular Sciences, Macquarie University, Sydney, NSW 2109, Australia; alexander.charles.carpenter@gmail.com (A.C.C.); ian.paulsen@mq.edu.au (I.T.P.); 2 CSIRO Synthetic Biology Future Science Platform, Canberra, ACT 2601, Australia

**Keywords:** biosensors, synthetic biology, analytics, molecular diagnostics, protein switches, aptamers, high-throughput screening, metabolic engineering

## Abstract

Biosensors are enabling major advances in the field of analytics that are both facilitating and being facilitated by advances in synthetic biology. The ability of biosensors to rapidly and specifically detect a wide range of molecules makes them highly relevant to a range of industrial, medical, ecological, and scientific applications. Approaches to biosensor design are as diverse as their applications, with major biosensor classes including nucleic acids, proteins, and transcription factors. Each of these biosensor types has advantages and limitations based on the intended application, and the parameters that are required for optimal performance. Specifically, the choice of biosensor design must consider factors such as the ligand specificity, sensitivity, dynamic range, functional range, mode of output, time of activation, ease of use, and ease of engineering. This review discusses the rationale for designing the major classes of biosensor in the context of their limitations and assesses their suitability to different areas of biotechnological application.

## 1. Introduction

In the past decade, the field of synthetic biology has flourished, significantly impacting fields such as metabolic engineering, protein engineering, computational biology, and whole-genome engineering [1]. A large portion of synthetic biology innovation has occurred under a framework of iterative cycles of “design-build-test” development. Growth in the field of synthetic biology can be correlated with innovations in each of the “design”, “build”, and “test” processes [2]. For example, there has been a significant effort in the standardization of parts within synthetic biology, with modularity and “plug and play” components receiving significant attention. Combined with the steady progress of systems biology, this modularization has enabled the “design” phase to become less time consuming and less dependent on specialized knowledge. The cost of DNA sequencing and synthesis has also decreased significantly in recent years, allowing the cheap synthesis of large constructs [3]. This has allowed a rapid improvement in the “build” phase, enabling researchers to explore a greater proportion of the biological solution space. Finally, high-throughput screening has also become a focal point within the synthetic biology “test” phase. The increased capacity for “design”, and “build” has led to an increased need for throughput in assessing the plethora of new designs. In part, this has been achieved with the incorporation of robotics and high-throughput analytics into the laboratory setting, wherein new designs can be assayed at a volume not possible for human researchers [4]. 

Biosensors represent a revolutionary new technology that can be implemented for high-throughput screening. They are most simply defined as an analytical tool comprised of biological components that are used to detect the presence of a target ligand and to generate a signal [5]. Biosensors are at the forefront of synthetic biology, both as a tool for high-throughput screening, but also as the direct result of advances within the synthetic biology field itself. Furthermore, biosensors have received increased attention as alternatives to traditional analytics due to the unparalleled specificity and sensitivity that biological parts afford compared to conventional analytical techniques.

Biosensor design and construction is a multidisciplinary endeavor and can require expertise from fields as diverse protein engineering, molecular biology, affinity chemistry, nucleic acid molecular dynamics, materials sciences, and nanotechnology. At their most basic, biosensors interface with a target ligand, undergo some form of change, and output a signal [5]. All of the parts of this process have a great diversity of possible configurations. Target ligands range from single atoms such as calcium [6], all the way through to whole proteins such as thrombin [7]. Output signals include processes as diverse as enzymatic activity, fluorescence, generation of electrical current, and transcriptional activity [5]. Equally as diverse are the mechanisms that transduce ligand detection into usable signals. The great diversity of biosensors is the focus of this review, with an exploration of their respective functionality, design rationales, limitations, and their applicability in a range of industrial and research contexts. 

## 2. Applications

Biosensors represent a significant step forward in the field of analytics. The incorporation of biological components in sensory diagnostics has begun to move analytics away from purely physics- or chemistry-based frameworks. This has allowed a tremendous diversity and specificity of biological components to perform analytical functions that are not well suited to conventional methods. The theoretical and demonstrated applications of biosensors span a significant breadth of human civilization and activity [5]. For the purposes of this review, biosensor applications will be divided and discussed in three broad categories based on their measurement scale.

### 2.1. Group Diagnostics: Environmental, Agricultural, and Industrial Applications

One application of biosensors is the periodic measurement of samples from a large group or area. One of the major applications in this category is quality assurance in food production. There is significant demand for biosensors that are capable of detecting a wide range of nutrients to aid in harvest-time optimization, as well as biosensors for detecting organic and inorganic contaminants [8]. Specifically, biosensors have been suggested for use in aquaculture for metabolite detection after fish death, to assess freshness and spoilage [9]. Biosensors have also been developed for monitoring of milk urea concentrations, and for the presence of *Escherichia coli* in drinking water [10,11]. Similarly, biosensors have been developed to monitor environmental samples such as soil, for the presence of explosive materials and industrial contamination [12]. Another major industrial application in this category is in bioprocess monitoring. This involves the sampling of large scale biological processes such as beer/wine fermentations, garbage degradation, or bio-remediation [13,14]. The current industry standards in these fields involve cumbersome laboratory based methods requiring sophisticated equipment and training such as polymerase chain reaction (PCR), mass spectrometry, and fluorescence in-situ hybridization, making the development of biosensors imperative [15]. The common themes in group diagnostic biosensor applications are that the frequency of sampling is periodic, turnaround time can vary be between several hours to several days, and detection does not necessarily need to be done “on site”. 

### 2.2. Point-of-Use Diagnostics: Medical, and Security Applications

The second major category of biosensor applications is for point-of-use diagnostics, which are characterized by the rapid and mobile detection of large numbers of individual samples. This is exemplified by biosensors used in medical diagnostics. For example, it has been suggested that a wide range of human diseases and pathogens could have biosensors developed for their detection [16]. This would decrease diagnosis and treatment times, enabling numerous lives to be saved [16]. This has been suggested for a range of cancer types and for the influenza virus [16,17,18]. Another major application in the point-of-use category is in security and defense. Screening packages, people, and vehicles for contraband and/or dangerous materials is an important process which could be streamlined through the use of biosensors [19,20]. Biosensor systems for some narcotics have already been employed in a micro-array format [21]. Similar systems are also used for the detection of 2,4,6-trinitrotoluene (TNT) [22]. Point-of-use diagnostic biosensors would have a significant advantage in diagnosis time, and ease of implementation compared to commonly used microscopy and real-time PCR-based techniques. Furthermore, unlike antibody based techniques, biosensor diagnostics could be more cost effective as they do not require animal sacrifice or mammalian cell culture-based protein production [23]. The unifying features of point-of-use diagnostic biosensors are that they need to have rapid response times, be mobile (preferably handheld), require low technical experience to operate, and be cost effective to mass produce and implement in high traffic environments (hospitals, transit hubs, and sporting events etc.). 

### 2.3. Single-Cell Diagnostics: Metabolic Engineering, and Synthetic Biology Applications

The final category of biosensor applications differs from the first two in that it involves detection at a very small scale. Rather than detecting a target ligand as an average from a large sample population or swab, single cell monitoring involves the detection of target ligands in each individual member within a cellular population. This is useful for high throughput screening, directed evolution, and control of gene circuits, as it can be used to identify and isolate individuals with desired phenotypes [24,25]. Advances in synthetic biology have seen an explosion in the capacity to generate diversity in metabolic pathways. However, the ability to efficiently sample that diversity has lagged behind. Biosensors offer a convenient method of assessing the concentration of a target ligand within each cell, allowing for the simultaneous screening of millions of genetic variants [13,26]. Another significant use of single cell diagnostic biosensors is as components in gene circuits. Biosensors can act as components in biological logic gates such as “and/or/not” gates, allowing the creation of complex synthetic regulatory networks with a wide range of applications [27,28]. A common theme of single cell diagnostic biosensors are that they need to be practically and economically applicable on a single-cell scale. That is, they need to be able to survey and respond to the target ligand concentration within individual cells without being impacted by other population members. This makes biosensors developed for single cell diagnostics significantly less tolerant to false positive/negative activation compared to other applications. 

## 3. Transcription Factor-Based Biosensors

Transcription factor based biosensors (TFBs) are protein-based biosensors that utilize cellular transcriptional and translational machinery to generate a signal in response to a target ligand. Target ligand association with the TFB protein results in transcriptional activity of a specific signal gene. TFBs were some of the earliest and most extensively developed biosensors. Indeed, transcription factor-based biosensors have been used for decades as part of genetic research [29,30]. This is likely due to the inherent modularity of promoter/operator sequences and their downstream transcribed genes. Furthermore, previous decades of genetic research have focused on transcriptional activation in response to environmental cues, providing a wealth of exploitable knowledge across a range of species. With the exception of a few recent cell-free systems, almost all TFBs function only in vivo [31,32].

### 3.1. Native Transcription Factor-Based Biosensors

#### 3.1.1. Function: Native Transcription Factor-Based Biosensors

**Figure 1:**
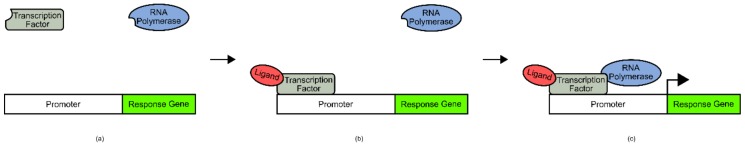
Transcription factor biosensor configuration. A known transcription factor protein that is regulated by a ligand of interest can be used to target a cognate promoter sequence that is used to initiate transcription of a response gene such as *GFP* or antibiotic resistance: (**a**) In the absence of ligand binding to the transcription factor, there is little or no recruitment of RNA polymerase to the promoter site; (**b**) When ligand is present at a concentration sufficient to bind transcription factor proteins, the transcription factor becomes localized to the promoter; (**c**) Recruitment of RNA polymerase then allows transcription of the response gene (e.g., *GFP* or antibiotic resistance).

Biosensors based on naturally occurring transcription factors are amongst the easiest to engineer, and therefore the most widely used. Crucially, these designs rely on the existence of a naturally evolved transcription factor for a ligand of interest. When a transcription factor is bound by its ligand, it can undergo a conformational shift or become localized to a promoter region to drive transcription/repression of a gene of interest (Figure 1). If the cognate promoter of a given transcription factor is known, it can be used to control the expression of a response gene that results in a range of signal outputs. Commonly used output signals include fluorescence, antibiotic resistance, increased growth rate, and bioluminescence.

Naturally occurring transcription factors have become a popular option in synthetic biology, used for a variety of metabolite detection systems [33] including para-hydroxybenzoic acid [34], NADP/NADPH [35,36] muconic acid [37], fatty acids [38], L-lysine, 3-hydroxypropionate [39,40], L-valine [41], malony-CoA [42], macrolides such as erythromycin [43], and putrescine [44]. 

#### 3.1.2. Design and Construction: Native Transcription Factor-Based Biosensors

Ligand-regulated transcription factors are abundant in nature [45,46,47], and can often be identified in the literature as being used for rapid deployment and testing as biosensors. After identification of a potential transcriptional regulator and promoter/operator pair, only simple genetic manipulation is needed to combine these elements with the desired signal output for the intended assay.

Construction is somewhat complicated when there is no known transcriptional regulatory elements which respond to the target ligand. This can sometimes be mitigated using dose-response transcriptomics or via insertional mutagenesis [30]. In these techniques, the host organism is exposed to increased concentrations of the target ligand, and genes which are upregulated in response are identified. The same process can then be repeated for compounds with slight functional differences to the target ligand. This can identify promoters that are up-regulated in response to the target ligand without being incorrectly activated by closely related compounds. The transcriptomic strategy has been successfully employed to identify a 1-butanol biosensor in *Saccharomyces cerevisiae* [48], and a farnesyl-diphosphate biosensor in *E. coli* [49]. 

#### 3.1.3. Limitations: Native Transcription Factor-Based Biosensors

One of the major limitations to this type of biosensor construction is the range of transcription factor promoter pairs available. Each species has a finite number of regulatory mechanisms through which it controls metabolic activity, and every metabolite does not have its own cognate transcription factor. Rather, it is common for only a few metabolites in a pathway to be involved in transcriptional feedback. 

The transcriptomic approach for promoter/operator identification can be useful; however, it is important to note some of the limitations of the regulatory mechanisms that it identifies. It is possible that the promoters/operators identified by this method will have off target activation from unforeseen sources. Unless this method uncovers a ligand specific transcriptional regulator, the resultant identified promoter/operator will only be the final downstream response of the cell to the ligand. That is, the transcriptome response may be specific to the target ligand when compared to other similar compounds, but that is no guarantee that unknown factors will not also induce the same response. Without a transcriptional regulator with a dedicated mechanism of ligand interaction, biosensors built in this way may have issues with specificity. This could become problematic if used in single cell diagnostics for directed evolution or high throughput screening, where off-target activation would result in the generation of false positives [24]. This kind of limitation is likely present in the butanol biosensors developed by Shi, et al. [48].

### 3.2. Heterologous Species Transcription Factor-Based Biosensors

#### 3.2.1. Function: Heterologous Species Transcription Factor-Based Biosensors

If transcriptional regulators for a target ligand do not exist in the desired host species, using transcriptional regulators from other species can be a valuable solution. This is important when assaying a ligand within a species that does not naturally produce or encounter said ligand. 

**Figure 2:**
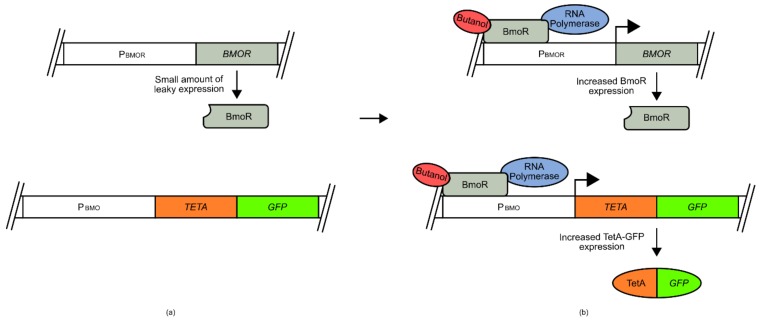
Butanol biosensor developed for use in E. coli. P_BMOR_ is upstream of the transcriptional regulator gene *BMOR*. P_BMO_ is upstream of a tetracycline resistance-green fluorescent protein fusion gene (*TETA-GFP*): (**a**) under low butanol concentrations, leaky expression driven by P_BMOR_ produces an insignificant amount of BmoR; (**b**) Increases in butanol concentrations allows coordination of BmoR to P_BMOR_ and P_BMO_. This allows recruitment of RNA polymerase and the expression of *TETA-GFP*, producing a cell survival and fluorescence signal. Additionally, by placing P_BMOR_ as the controller of *BMOR* transcription, this signal is self-amplifying.

For example, the development of a butanol biosensor for use in *E. coli* made use of a σ factor and promoter sequence from the species *Thauera butanivorans* (Figure 2) [50,51]. The promoter sequence (P_BMO_) was placed at the 5 prime end of a tetracycline resistance *TETA*-*GFP* fusion gene, while the σ factor (*BMOR*) was expressed under the BMOR promoter [50]. Both P_BMO_ and P_BMOR_ are activated by BmoR upon binding of butanol. Under conditions of low butanol, limited TetA-GFP fusion protein was produced, as with the BmoR sigma factor. However, under increased butanol concentrations, the BmoR sigma factor increased expression of TetA-GFP and of itself as part of a signal amplification process. This created an amplifying signal that allowed the selection of cells that had increased internal butanol concentrations using fluorescence activated cell sorting (FACS) or cell survival.

**Figure 3:**
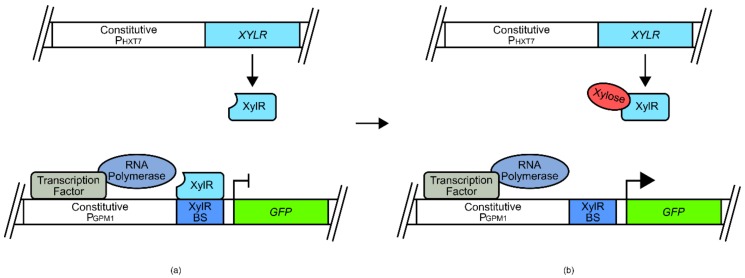
Xylose biosensor developed for use in *S. cerevisiae*. The *XYLR* gene is under the regulatory control of P_HXT7_. *GFP* is under the regulatory control of P_GPM1_ with an inserted XylR binding site (BS); (**a**) Under low xylose concentrations, *XYLR* is constitutively expressed by P_HXT7_, and binds to the XylR BS, inhibiting the ability of RNA polymerase and other transcriptional machinery from transcribing the downstream *GFP* gene. (**b**) Under increased xylose concentrations, the XylR protein binds xylose, disrupting its ability to associating with XylR BS. Free of impediment, this allows P_GPM1_ to induce the transcription of *GFP*. Thus, increases in xylose concentrations correlate with an increase in GFP signal.

Sometimes the transplantation of transcription factors from another species may not be viable, especially if it is attempted across domains of life. This is because prokaryotes and eukaryotes have different transcriptional activation systems that diverged long ago in evolutionary history. This means that a prokaryotic transcriptional regulator may not interface with eukaryotic transcription machinery, even if it is bound at a promoter region and activated by a ligand. Alternative approaches have therefore been employed to circumvent some of these problems. Whilst it is difficult to engineer prokaryotic transcription factors to directly initiate transcription in a eukaryotic chassis and vice versa, success has been found with transcriptional repressors. For example, several xylose biosensors were developed for use in *S. cerevisiae* using the xylose binding transcriptional repressor XylR and its corresponding binding sequence from bacteria [52]. The XylR binding sequence was placed adjacent to either/both the natural *S. cerevisiae* P_GPM1_ upstream activation sequence (UAS) or the TATA box (Figure 3). Under normal conditions, the XylR transcriptional repressor binds to its cognate operator sequence, inhibiting the binding of transcription factors at the UAS site, or inhibiting the recruitment of transcriptional machinery at the TATA box site of P_GPM1_. However, when xylose concentrations increase, this competitively binds to the XylR transcriptional repressor, reducing coordination to its operating sequence, allowing native regulation of P_GPM1_ to occur, and resulting in transcription of a reporter *GFP* gene [52]. 

#### 3.2.2. Design and Construction: Heterologous Species Transcription Factor-Based Biosensors

With sufficient available literature, importing a heterologous biosensor can be a relatively straightforward process. It involves codon optimization of the identified transcription factors, and biosensor tuning by modulating promoter length/binding domain lengths and copy number. In the butanol biosensor example mentioned above, transplanting the BmoR σ factor into *E. coli* worked because it was elucidated that BmoR did not require any activation signals other than the binding of butanol (i.e., an additional sensory histidine kinase as is typical of most σ factors) [50]. As both *T. butanivorans* and *E. coli* are prokaryotes, recruitment and initiation of RNA polymerase activity was likely to be compatible. 

Compared to transcriptional activation, transcriptional inhibition can be implemented using somewhat simpler design principles. Indeed, the use of transcription inhibition as part of biosensor systems has been done for many years, with some of the earliest versions being used for gene function research [53]. Development of transcriptional inhibition based biosensors first involves identifying a transcriptional regulator that responds to the compound of interest in a prokaryotic organism. It is then necessary to identify the operator sequence with which the repressor binds the DNA. This operator sequence can then be placed within a native eukaryotic promoter near the TATA box or the upstream activating sequence [52]. Binding of the compound of interest to the repressor protein inhibits binding to the repressor operator sequence within the *S. cerevisiae* promoter, allowing transcription. If this architecture is placed upstream of a fluorescent protein or an antibiotic resistance gene, then the presence of the compound of interest will decrease fluorescence/antibiotic resistance. This process was implemented in the development of the XylR-based xylose biosensors [52]. Three XylR transcription factors from the bacterial species *Staphylococcus xylosus*, *Bacillus subtilis*, and *Bacillus licheniformis*, were already well-known and characterized, as were their corresponding DNA binding sites. These binding sites were inserted into a simplified P_GPM1_ promoter which controlled the expression of a *GFP* gene. The sites were chosen due to their proximity to either/both the P_GMP1_ UAS or TATA box. This method of biosensor creation was largely successful, with 11 of the 12 constructed designs showing a significant signal upon detection of xylose [52].

#### 3.2.3. Limitations: Heterologous Species Transcription Factor-Based Biosensors

The use of heterologous transcription factors as the basis for biosensor development is not without some limitations. Construction is generally reliant on known transcriptional activators with well-characterized mechanisms. Transcriptional regulation is rarely a simple process, and the recruitment of transcriptional machinery often relies on several regulators and co-activators. Unlike native transcription-factor based biosensors, construction of heterologous transcription factor-based biosensors requires detailed knowledge on all the regulatory components of the system so that the regulatory pathway can be rationally transferred into the host chassis. This is especially true when trying to construct biosensors using transcription factors from other domains of life where even the most basic transcriptional machinery has significant underlying incompatibilities. Even when there is significant knowledge on transcriptional activation for a target compound in one species, this does not necessarily mean that the regulatory system will be amenable to transposition. Signaling that is based on pathways which involve large cascades, the recruitment of many co-activators, or the involvement of membrane proteins, can significantly increase the difficulty of transposing a transcriptional activation system from one domain of life to another. Finally, as with native transcription factor based biosensors, the pool of heterologous transcription factors available for use in target compound biosensor construction is finite. If responsive transcription factor promoter pairs are not known, or cannot be identified via transcriptomic analysis, then there is little re-course for biosensor construction using these methods.

### 3.3. Modular Transcription Factor-Based Biosensors

#### 3.3.1. Function: Modular Transcription Factor Based Biosensors

A potentially more versatile method of engineering TFBs involves the use of modular protein domains to induce transcription in response to a target ligand. The critical components in this type of biosensor construction are protein domains that dimerize/co-ordinate with each other in the presence of a target ligand. These domains can then be expressed separately as linker fusions to well-characterized transcriptional activation domains and DNA binding domains (DBDs). In the absence of the ligand, these domains are separated so that there is limited transcription of the output gene. In the presence of the target ligand the domains are co-localized at the promoter so that transcription can be initiated. 

**Figure 4:**
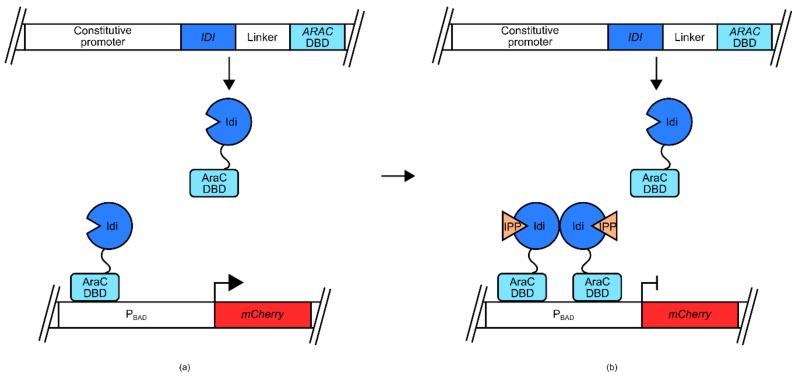
Isopentenyl-pyrophosphate (IPP) biosensor for use in E. coli. An IPP isomerase (Idi) domain-tether-AraC DNA binding domain fusion protein was expressed under the regulatory control of a constitutive promoter. Whilst an *mCherry* gene was under the regulatory control of P_BAD_: (**a**) Under low IPP concentrations, Idi-linker-AraC DNA binding domains (DBD) fusion protein associated with P_BAD_ and the AraC DBD induced transcription of mCherry; (**b**) Under increased IPP concentrations, Idi-mediated fusion protein dimerization inhibited the ability of AraC DBD from efficiently inducing transcription of *mCherry*. Thus, increases in IPP concentration were correlated with decreases in the mCherry signal.

This approach was utilized recently to engineer isopentenyl-pyrophosphate (IPP) biosensors for use in *E. coli*, and *S. cerevisiae* [54]. The *E. coli* version of this biosensor used the *araBAD* promoter (P_BAD_) and its native transcriptional regulator AraC (Figure 4). In its native context, the AraC transcriptional regulator binds arabinose, resulting in a conformational change that allows association with P_BAD_ and induction of transcription. In the mentioned biosensor, the DNA binding domain of AraC is joined via a linker to a known IPP-binding domain, IPP isomerase (Idi). Due to crystallographic data, it was suspected that Idi would form a dimer in the presence of IPP. At low IPP concentrations, the AraC DBD-Idi fusion protein is free to bind to P_BAD_, inducing transcription of a mCherry-encoding reporter gene. However, as concentrations of IPP increase, the IPP isomerase domains dimerize, blocking the ability of the AraC DBD from inducing transcription from P_BAD_, and reducing the expression of mCherry.

**Figure 5:**
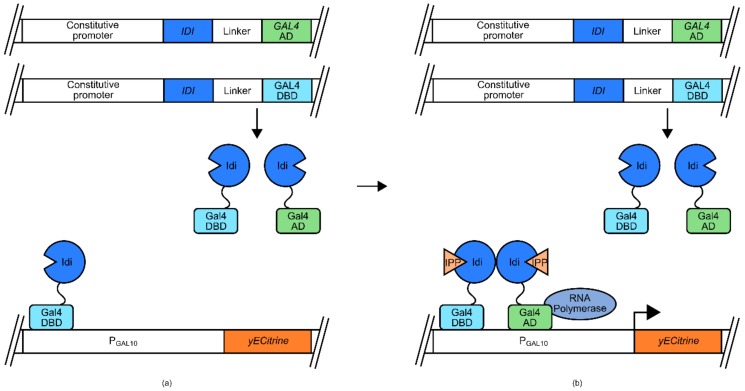
Isopentenyl-pyrophosphate (IPP) biosensor for use in *S. cerevisiae*. Two fusion proteins were used in the biosensor. The first was IPP isomerase (Idi)-tether-Gal4 activation domain (Gal4 AD). The second was IPP isomerase (Idi)-tether-Gal4 DNA binding domain (Gal4 DBD). Both fusion protein genes were under the regulatory control of constitutive promoters. Also used in this biosensor was a *yECitrine* gene under the control of P_GAL10_: (**a**) Under low IPP concentrations, the fusion proteins were expressed, but had no effect on *yECitrine* expression; (**b**) Under increased IPP concentrations, the two fusion proteins are able to dimerize via the Idi domains. Gal4 DBD localized the dimer to P_GAL10_, while Gal4 AD induced the transcription of *yECitrine*. Thus, increases in IPP concentration were correlated with increases in the yECitrine signal.

The ability of IPP isomerase to dimerize was also used in the development of an IPP biosensor for use in *S. cerevisiae* [54]. In this instance the separate DBD and activation domains (AD) of the transcription factor Gal4 were tethered to IPP isomerase domains (Figure 5). Also utilized in this design is a GAL promoter (*P_GAL10_*), controlling the expression of a responsive *yECitrine* gene for fluorescent marker signal generation. At low concentrations of IPP, the Gal4 DBD-IPP isomerase fusion protein is able to associate with P_GAL10_, but has no effect on transcription. However, increasing concentrations of IPP result in the dimerization of the IPP isomerase domains, bringing the Gal4 AD into close proximity of P_GAL10_ inducing transcription of *yEcitrine*. 

#### 3.3.2. Design and Construction: Modular Transcription Factor-Based Biosensors

In the examples discussed above, natural transcription factors for IPP had not been discovered prior to construction. Instead, both of these biosensors utilized ligand binding domains that were suspected to dimerize in the presence of a compound of interest. The construction process as a whole was fairly straightforward in both examples. As both designs used modular components, there was only a small amount of rational design that was necessary to generate functional biosensors in each case [54]. The two major pieces of construction work in these designs involved confirmation that the IPP isomerase domains dimerized as predicted in response to IPP, and then choosing the linking domains that would be most suitable to connect the components [54]. After construction, optimization of the *E. coli* biosensor was performed by subjecting the AraC DBD-IPP isomerase fusion protein to error-prone PCR mutagenesis, generating 60 versions of the sensor [54]. Biosensor variants with desirable characteristics were then selected from the mutant library using sensor output in response to the stimulus as selection. The *S. cerevisiae* version was also expanded upon, with two alternatives being created. This involved swapping out the IPP isomerase domains for either Idi1, or Erg20 domains, both of which are known to use IPP as substrates in their respective catalyzing reactions [54]. 

#### 3.3.3. Limitations: Modular Transcription Factor-Based Biosensors

The great advantages provided by modular transcription factor based biosensors are intertwined with some of their largest limitations. Modularity makes construction easier; however designing/finding modular domains that can be used across a range of biosensor configurations has their own limitations. Generic transcriptional activation domains and generic DNA binding domains are simple enough to obtain. However, finding/creating ligand binding domains that either dimerize or produce a useful conformational change upon ligand association can be more difficult, and begins to move construction away from simple genetic manipulations into more complicated protein engineering. Despite this, modular transcription factor biosensors show great promise for the detection of ligands for which natural transcription factors do not exist. To enhance this technology would require that binding proteins/peptides be generated using random selection techniques such as phage/yeast display or a two-hybrid system [55,56].

### 3.4. Advantages and Applicability: Transcription Factor-Based Biosensors

#### 3.4.1. Group Diagnostics: Environmental, Agricultural, and Industrial Applications

Transcription Factor-Based Biosensors (TFBs) obviously have inherent limitations regarding response time. However, with sufficient literature on transcription factors, they can also be some of the easiest biosensors to design/construct. Thus, their utility in group diagnostics is highly dependent on the time scale necessary for measurement. As such, TFBs have demonstrated significant applicability in environmental diagnostics [45,57]. One other area where TFBs excel in group diagnostics is in bioprocess control. Being one of the natural regulatory mechanisms of the cell, large scale bioprocess responses can be feasibly coupled to biosensor activation in simple gene circuits. For example, TFBs that detect glucose, urea, and unfolded protein responses have been implemented previously to optimize large scale fermentations [58].

#### 3.4.2. Point-of-Use Diagnostics: Medical, and Security Applications

The major limitation in using TFBs for point-of-use diagnostics is response time. The requirement for living cells to be cultured means that specialized equipment and expertise are required for operation. Furthermore, biosensor output requires the time necessary for cell growth, transcription, translation, and protein folding/maturation. These factors mean that other biosensor types are more suitable for high throughput point-of-use diagnostic applications. 

#### 3.4.3. Single-Cell Diagnostics: Metabolic Engineering, and Synthetic Biology Applications

A major advantage that TFBs present is a natural interface with transcriptional output within a single living cell. The fact that transcription and translation of output proteins can occur over time in an additive manner means that this class of biosensor has a natural capacity for signal amplification, making them highly sensitive to changes in ligand concentration. These features make TFBs widely applicable to metabolic engineering applications [59,60]. For example, they can be used to report on the productivity of individual metabolites within each cell in an engineered or mutated population so that superior producers can be evolved using a survival output gene, or so that libraries of genetic parts can be screened using FACS [24,26]. In addition to these invaluable functions, transcription-factor biosensors can also be used to autonomously and dynamically regulate gene expression in response to endogenous metabolite production levels so that optimal fluxes can be maintained and the build-up of toxic compounds is avoided [49].

## 4. Nucleic Acid-Based Biosensors

Nucleic acid based biosensors are built using primarily nucleic acids as functional components. These are DNA or RNA sequences that have binding affinity to a target ligand. The binding of the target ligand to the nucleic acid sequence alters its structure which is utilized via a variety of mechanisms to generate an output signal. 

### 4.1. Aptamers

#### 4.1.1. Function: Aptamers

Aptamers are single-stranded DNA or RNA molecules that have affinity to a target ligand [61,62,63]. In general, the binding of the DNA/RNA aptamer to the target ligand induces a conformational change in the aptamer. For some, this change can involve transitioning from a linear to a more stem-looped structure, or from a hair-pin to a more ligand-coordinated structure. It is the changes in the secondary and tertiary structures that are induced upon binding to a target ligand that are used to generate a signal. While this review does not provide a comprehensive catalogue of all of the possible aptamer biosensor signal generation methods, two illustrative examples will be explored (for more detail on possible outputs of aptamer biosensors please see Song, et al. [64]).

**Figure 6:**
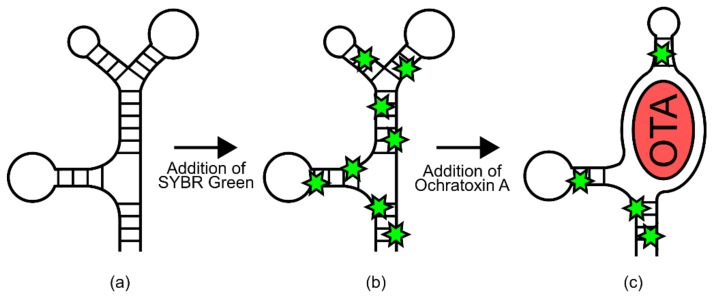
Ochratoxin A biosensor. The biosensor is comprised of a DNA aptamer for Ochratoxin A (**a**) aptamer with no added SYBR Green or Ochratoxin A; (**b**) coordinated with SYBR Green (green star); (**c**) and coordinated with OTA (red oval) and SYBR Green. Coordination of OTA to the aptamer results in decreased binding sites for SYBR Green, and therefore decreased fluorescence. Figure adapted from McKeague, et al. [65].

In one example, a known DNA aptamer for Ochratoxin A (OTA) was thought to have a predominantly stem-looped structure that was disrupted via ligand binding (Figure 6) [65]. As such, an assay was employed in which signal generation relied on the double-stranded DNA-binding dye SYBR Green. Without OTA, the aptamer would form a stem-loop rich structure, providing many binding sites for SYBR Green [65]. However, upon exposure to OTA, this structure would be disrupted, decreasing SYBR Green binding and therefore fluorescence [65]. This resulted in a biosensor with a linear response to OTA between 9 nm–100 nm [65]. 

**Figure 7:**
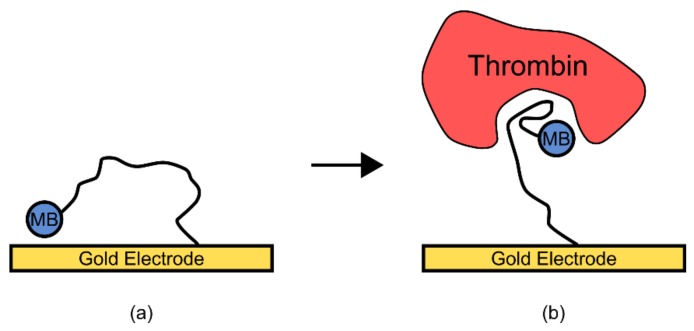
Thrombin biosensor. Thrombin-binding aptamer immobilized on a gold electrode with covalently added methylene blue group (MB). (**a**) In the absence of thrombin, the flexible aptamer structure allows the MB group to contact the electrode, donating an electron and generating a signal. (**b**) Addition of thrombin stabilizes secondary structure, reducing contact of the MB group with the electrode, reducing signal generation. Figure adapted from Xiao, et al. [7].

In a second example, an aptamer for the protein thrombin was previously thought to have a very unstable structure in its unbound state but formed stable secondary and tertiary structures upon ligand binding [7]. To engineer a readable output from this aptamer, a methylene blue group was covalently added, and the aptamer was immobilized on a gold electrode (Figure 7) [7]. In the unbound state, the lack of structure in the aptamer allowed the added methylene blue group to randomly collide with the gold electrode, transferring an electron. However, upon thrombin binding the aptamer was thought to have adopted a more rigid structure, reducing electron transfer and thus removing the signal [7]. This resulted in a biosensor which was sensitive enough to cover the normally observed physiological concentrations of thrombin [7].

#### 4.1.2. Design and Construction: Aptamers

**Figure 8:**
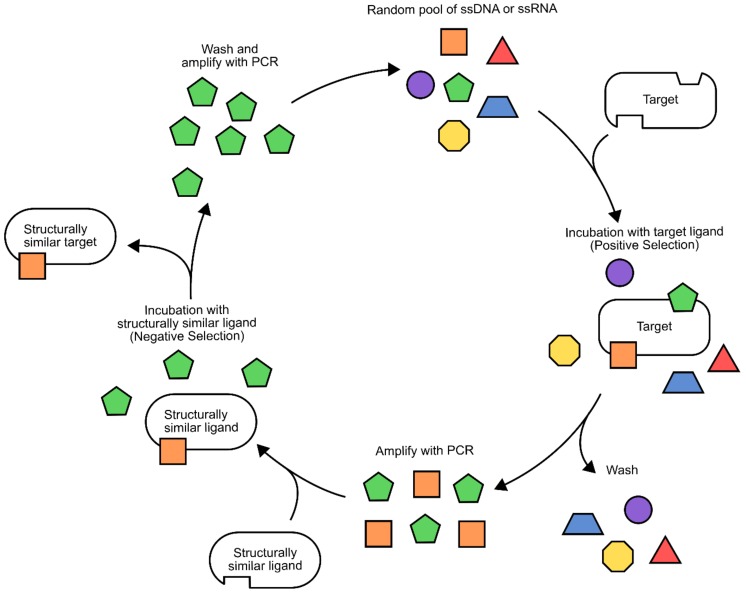
The systematic evolution of ligands by exponential enrichment (SELEX) process. The SELEX process uses a series of incubation and wash steps to identify single-stranded (ss) DNA or ssRNA molecules with binding affinity to a target ligand. Positive and negative selection steps are employed to avoid binding affinity to structurally similar compounds. Polymerase chain reaction (PCR) is used to amplify positive sequences and enrich them as a proportion of the total population.

The most constraining factor in aptamer biosensor development is the identification of single-stranded DNA/RNA sequences that bind specifically to the target ligand. The process used for this is known as systematic evolution of ligands by exponential enrichment (SELEX) [61,62,63]. Although there are many variations on the process, a general workflow will be presented here (Figure 8) [66,67] (for a more comprehensive review of different SELEX methodologies, please refer to [67]). To begin with, a random library of 30–80 bp nucleotides, flanked by known sequences for PCR amplification, is generated. Next, the target ligand and library members are incubated together, then washed to remove non-binding sequences. It is often necessary to immobilize small target ligands during this step to ensure the removal of non-binding sequences. Alternatively, larger ligands and their bound aptamers can be isolated using centrifugation [68]. The bound sequences are then liberated from the target ligand and amplified using PCR. This biases the library composition towards sequences that have affinity to the target ligand. This process can be repeated with increasing stringency to identify sequences with the highest binding affinity [67,69,70]. 

After identification of a ligand-binding aptamer, two construction methodologies are predominantly employed to link ligand binding to a signal output. The first uses a combination of modelling techniques to rationally design a signal output based on the known folding of the identified aptamer in the presence of the target ligand [71]. However, this requires a level of structural information, which may not be available. The second approach involves taking a larger pool of identified target ligand binding aptamers and building a suite of aptamer biosensors. Correct function of these biosensors can then be assayed using signal generation in response to the target ligand as a way to identify useful biosensor designs.

#### 4.1.3. Limitations: Aptamers

One of the major limitations of aptamer-based biosensors is how the SELEX process is applied to small molecules. This is because unlike protein targets, small molecule targets need to be immobilized prior to incubation with the aptamer library [70]. Immobilization is frequently done with agarose, sepharose, or magnetic beads, and allows non-binding aptamer sequences to be removed from the population [69]. The first issue raised by this method is that in order to immobilize a small molecule, it requires a functional group through which conjugation can occur. For many small molecules this may be one of few residues that could facilitate aptamer binding, and by using it for conjugation, it can no longer be used as a binding site. Furthermore, many small molecules do not possess a residue that is amenable for conjugation. In these circumstances, chemical modification is necessary to allow immobilization. This results in aptamers being screened against variants of the target ligand and not the native target itself, causing a lack of binding specificity [70]. Finally, in both of the immobilization methods mentioned, even when attempting to control for these issues, it is quite common for identified aptamers to have some binding affinity for the solid support matrix itself [67]. 

Aptamer-based biosensors are fairly simple structures compared to protein based biosensors. Whilst this is an advantage for the SELEX process, it also means that there can be issues with affinity and specificity [72]. The restricted sequence size and number of natural nucleotides available for aptamer development can result in unintended activation from structurally similar compounds [70,72,73].

Another limitation that aptamer-based biosensors face is that their three dimensional structures are highly influenced by the surrounding conditions, such as pH and temperature [67]. Considerations can be made during the SELEX process to mimic the intended assay environment to find the most suitable aptamers. However, this can make compatibility across assay systems difficult, and this could mean that an aptamer developed for in vivo detection of a target ligand may not be functional in vitro. Additionally, it may be that an aptamer developed for the detection of a target ligand in the cell lysate of one species may not work with another [65,67].

### 4.2. Riboswitches

#### 4.2.1. Function: Riboswitches

Riboswitches are a class of biosensors made of single-stranded RNA [73,74,75,76]. Conceptually, they can be thought of as an extension on aptamer-based biosensor technology. Riboswitches are comprised of two joined RNA domains, the first is an aptamer that binds to the target ligand, and the second is a response domain that is used to generate a signal after ligand binding [73,74,75,76]. What separates riboswitches from the described aptamer-based biosensors is that the response domain is an extension of the aptamer RNA sequence [73,76]. The response domain and aptamer domains normally have a specific secondary structure that is strongly influenced by complementary base-pair binding between the two domains [74,75,76]. The binding of the ligand to the aptamer domain then induces a conformational change in secondary structure that influences the structure of the response domain, thereby generating a signal [74,75,76]. Frequently, the response domain is a messenger RNA (mRNA) transcript, and the change in structure either allows or restricts translation from taking place. However, other signal outputs are possible [74,75,76].

**Figure 9:**
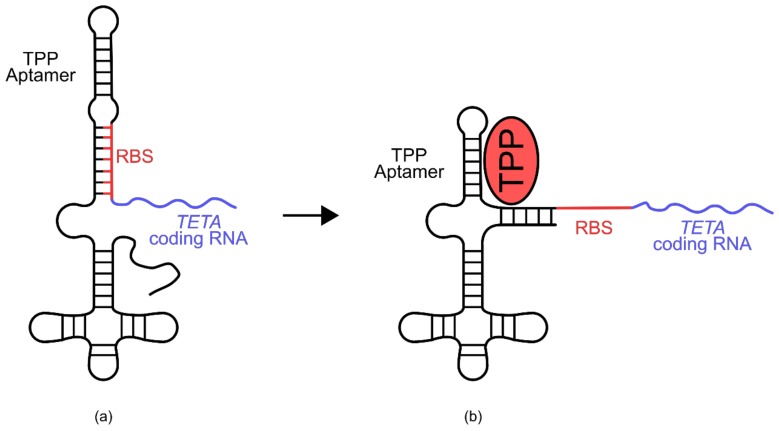
Thiamine pyrophosphate (TPP) biosensor. The biosensor is comprised of a TPP-binding aptamer, a *TETA*-coding RNA, and a modified spacer sequence containing the ribosomal binding site (RBS): (**a**) Biosensor without TPP. Base pair homology keeps the RBS inaccessible to the ribosome, preventing translation of the *TETA*-coding RNA; (**b**) TPP biosensor coordinated to TPP. Coordination allows a more energetically favorable secondary structure to be adopted, which liberates the RBS, allowing translation of the *TETA*-coding RNA. Under increased TPP concentration conditions, *E. coli* cells containing this biosensor are able to grow in the presence of the antibiotic tetracycline. Figure adapted from Muranaka, et al. [77].

An example of such a biosensor is the thiamine pyrophosphate (TPP) riboswitch developed for use in *E. coli* (Figure 9) [77]. In this sensor, a known TPP-binding aptamer sits 25 nucleotides upstream of the ribosomal binding site of an RNA transcript encoding tetracycline resistance (*TETA*) [77]. Part of the TPP aptamer portion of this riboswitch shares homology with the ribosomal binding site and several adjacent residues. After transcription, the aptamer domain folds back onto the ribosomal binding site and blocks access of the ribosome, preventing translation [77]. In the presence of TPP however, the TPP aptamer preferentially associates with the ligand, liberating the ribosomal binding site and allowing translation of the *TETA* coding transcript. When exposed to elevated TPP concentrations, *E. coli* cells containing this biosensor are able to grow in the presence of tetracycline. 

**Figure 10:**
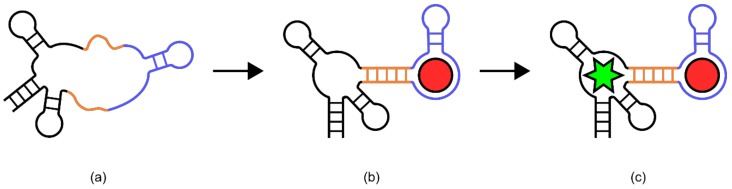
General Spinach based biosensors. (**a**) The Spinach aptamer (black) is disrupted with linker sequences (orange) and a ligand binding aptamer (blue), inhibiting its ability to bind the fluorophore 3,5-difluoro-4-hydroxybenzylidene imadazolinone (DFHBI). (**b**) Coordination of the target ligand (red circle) to the ligand binding aptamer provides sufficient stability to allow the linker regions to assemble. This in turn allows the Spinach aptamer to form its native secondary structure. (**c**) The reformation of the Spinach aptamer allows the coordination and activation of DFHBI (green star), resulting in ligand dependent fluorescent signal generation. Figure adapted from Paige, et al. [78].

A series of in vitro and in vivo riboswitches have also been made based on a known aptamer called [78] “Spinach” [78]. The Spinach aptamer was known to form a stem-looped structure in complex with the fluorophore 3,5-difluoro-4-hydroxybenzylidene imadazolinone (DFHBI), producing a fluorescent signal [78]. The riboswitch biosensors described here used ligand binding aptamer domains linked to the spinach aptamer through “transducer” sequences (Figure 10) [78]. These transducer sequences were short and comprised primarily of weak base pair interactions, such that the formation of a stable Spinach stem loop structure was unlikely [78]. However, binding of the ligand to the ligand binding aptamer provided enough stability in the transducer sequences to allow the spinach aptamer to adopt its native structure. This allowed binding of DFHBI and production of a fluorescent signal [78]. Biosensors using this design were constructed for adenosine diphosphate (ADP), S-adenosyl methionine (SAM), guanine, and guanosine 5-triphosphate, showing changes in fluorescence upon ligand binding of 20, 20, 25, 32, and 15-fold respectively [78]. 

Although it will not be discussed in detail, theophylline biosensors were recently developed by aptamer incorporation into a CRISPR Cas9 guide RNA [79]. Incorporation of an aptamer domain into the guide RNA was shown to be able to either stabilize or disrupt correct guide RNA structure in response to the target ligand [79]. This switching of guide RNA structure was then used to alter the targeting of de-activated Cas9 (dCas9). De-activated Cas9 binding to the upstream regions of reporter genes allowed inhibition of transcription of reporter genes [79]. Together, this enables ligand-dependent repression or de-repression of reporter genes. Although relatively new, this technique could have significant impacts on riboswitch-based biosensor development, as it allows for regulation at the transcriptional level, as opposed to at the conventional translational level. Furthermore, use of the CRISPR Cas9 system implies that biosensors developed in this way could be applied in a multiplex fashion, controlling multiple gene outputs simultaneously. 

#### 4.2.2. Design and Construction: Riboswitches

The TPP aptamer and *TETA* gene had previously been investigated prior to assembly, however the two had never been used in conjunction. The limiting factor in this design was identifying/building a response element that would allow translation after the binding of TPP to its aptamer, but that would repress translation when unbound. To do so, mutagenesis and a high throughput screening processes was employed [77]. A library was generated in which a variable number of random nucleotides, were inserted between the TPP aptamer and the ribosome binding site. To select for functional biosensor activity, *E. coli* cells expressing members of this library were grown in media containing ampicillin and TPP [77]. Cells that contained a biosensor with the desired characteristics would detect TPP and translate the *TETA* transcript, endowing resistance to ampicillin. A negative selection screen was also used to remove biosensors variants, which resulted in constitutive translation of the *TETA* transcripts [77].

Construction of the spinach-based riboswitches mentioned above was done using pre-existing aptamer domains for both DFHBI and the ligands of interest [78]. The majority of the engineering in these examples involved developing transducer sequences connecting the two known aptamers [78]. Unlike the example of the TPP riboswitch, the construction of spinach-transducer sequences was a mostly rational process using computational modelling to design a few possible configurations before testing viability [78].

It should be noted that although the examples listed above used pre-existing or natural aptamer domains, a large part of riboswitch research has utilized non-natural/synthetic aptamer domains. These can be natural aptamers that have undergone mutagenesis and selection to alter ligand binding specificity, or aptamers generated using the previously discussed SELEX process [80,81].

#### 4.2.3. Limitations: Riboswitches

The main limitation of riboswitch technology is its ability to integrate aptamer domains with response domains to generate functional riboswitches. Both natural and SELEX-identified aptamers have strong and specific binding to their ligands of interest. However, progressing from aptamer identification through to a functional synthetic riboswitch is not a simple process. In order to utilize most modelling techniques, the precise nature of binding to the target ligand needs to be known [82]. This is necessary, as a conformational change needs to be propagated from the aptamer domain through to a signal generation mechanism [82]. Whilst this seems simple, in reality, the criteria that are necessary for identifying which aptamers will make good riboswitches are not fully understood [74,83]. Alternatively, a mutagenesis and selection methodology can be used to integrate aptamers with response domains, requiring significantly less structural information/modelling [77].

### 4.3. Advantages and Applicability: Nucleic Acid-Based Biosensors

#### 4.3.1. Group Diagnostics: Environmental, Agricultural, and Industrial Applications

Nucleic acid based biosensors are well suited to group diagnostic applications. The ability of the SELEX process to identify novel binding aptamers means that biosensors can be developed for a wide range of applications. The success of this process, especially in identifying aptamers for protein targets, suggests that it could be applied for a range of food safety applications [67]. As the frequency and mobility of testing are usually less demanding in group diagnostics, the requirements for biosensors are also somewhat more relaxed than point of use or single cell diagnostic biosensors. Thus, the relatively simple fluorescent outputs of most nucleic acid based biosensors are compatible with the time scale and location requirements of most group diagnostic applications. 

#### 4.3.2. Point-of-Use Diagnostics: Medical, and Security Applications

Due to the high volume of individual samples involved in point-of-use diagnostic biosensor monitoring, some of the primary considerations in their development are speed, cost, and mobility. As such, a large area of focus in point-of-use diagnostic biosensor development has been placed on generating biosensors that can interface electrochemically with small electronic devices. The majority of aptamer and riboswitch biosensor designs do not fall into this category, with the notable exception of electrochemical aptamer biosensors [84]. These biosensors can produce an electrical signal induced by ligand binding to the aptamer [84,85]. An important consideration however, is that at this stage a large proportion of developed electrochemical aptamer biosensors require a two-step incubation process, increasing diagnostic time, and are designed for protein targets rather than small molecules, due to the limitations mentioned above [7,84,86].

#### 4.3.3. Single-Cell Diagnostics: Metabolic Engineering, and Synthetic Biology Applications

As producing, purifying, and transforming whole biosensor components into individual cells is costly and time consuming, the majority of single-cell biosensing is focused on biosensors that can be genetically encoded. While this is difficult for single-stranded DNA aptamers, it has been achieved for single stranded RNA aptamers [78,87]. These RNA aptamers rely on a fluorescent output, which can then be visualized on a single cell basis e.g., fluorescence-assisted cell sorting. Moreover, riboswitches have been employed successfully as genetically encoded in vivo biosensors. Their ability to be encoded within messenger RNA (mRNA) transcripts, effecting downstream gene expression, allows riboswitches to trigger cell survival phenotypes and function as parts of gene regulatory circuits.

## 5. Transcription-Independent Protein-Based Biosensors

Transcription-independent protein-based biosensors (TIPBs) are the most diverse class of biosensors in terms of mechanisms of detection and signal outputs. For the purposes of this review, TIPB designs have been divided into three broad categories that span a wide range of complexity. The driving factors behind the differences in complexity are due to the quality/quantity of background knowledge and the challenges involved in protein engineering. Key components of a TIPB include both receptor and response domains. The receptor domain is involved in interacting with the ligand of interest and the response domain is responsible for converting that interaction into a usable output. 

### 5.1. Integrated Transcription-Independent Protein-Based Biosensors

#### 5.1.1. Function: Integrated Transcription-Independent Protein-Based Biosensors

“Integrated TIPBs” refers to biosensors in which receptor domain/s for a target ligand are expressed as a fusion protein with little to no linker domain to a response domain. In these cases, target ligand binding to the receptor domain/s generates a conformational change that is directly transmitted to the response domain/s, inducing signal generation [88]. This type of design most closely mimics natural enzymes/signaling proteins generated through evolution via domain recombination [89]. 

**Figure 11:**
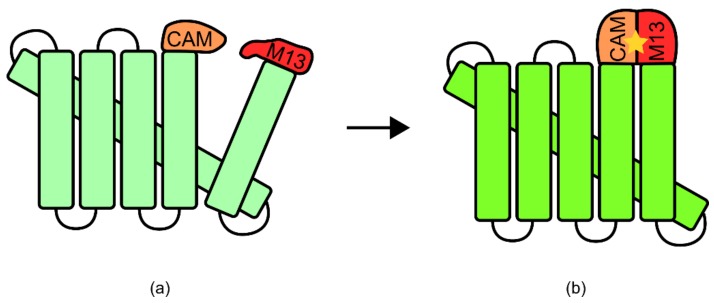
Calcium biosensor. This biosensor is one fusion protein comprised of a cyclically permuted green fluorescent protein (GFP), and calmodulin (CAM), and M13 domains: (**a**) At low calcium concentrations, the CAM and M13 domains do not coordinate efficiently, disrupting the structure of the cyclically permuted GFP. This disruption reduces the fluorescent output of the protein. (**b**) Upon binding of calcium (yellow star), the CAM and M13 domains form a more compact and coordinated configuration, allowing the GFP to return to its natural state, increasing fluorescence. Figure adapted from Nagai, et al. [90].

One well-known example of an integrated TIBP is the calmodulin and green fluorescent protein (GFP)-based biosensor for calcium. In this biosensor, a circularly permuted GFP was expressed with both calmodulin and M13 domains known for associating with calcium (Figure 11) [90] (for more information regarding circular permutation please see Yu and Lutz [91]). While unbound, these domains were disordered, disrupting the native structure of GFP [90]. However, upon binding of calcium to both domains, they adopted an ordered and compact conformation, allowing GFP to fold correctly and to produce a fluorescent signal [6,90].

**Figure 12:**
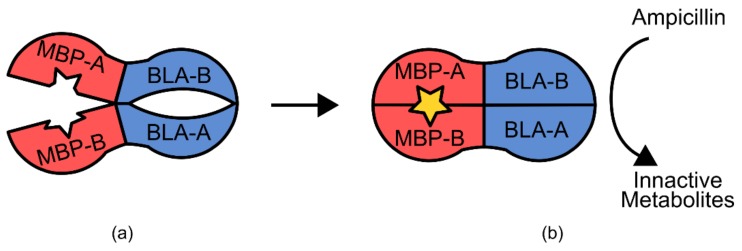
Maltose Biosensor. This biosensor is one fusion protein comprised of a maltose-binding peptide (MBP) and a β-lactamase enzyme (BLA), each of which has been split into two portions (A and B): (**a**) In the absence of maltose, the MBP-A and MBP-B domains exist in an open hinge conformation, disrupting the structure of the fused BLA-A and BLA-B domains. (**b**) Upon binding of maltose (star) to the MBP-A and MBP-B domains, this hinge closes, bringing the BLA-A and BLA-B domains back into the correct position. This reconstitutes the enzymatic activity of the BLA protein, allowing cells expressing this biosensor to grow in the presence of β-lactam antibiotics as a signal output.

Another example is the maltose biosensor, which uses an *E. coli* maltose-binding protein and a circularly permuted β-lactamase gene [92]. The maltose binding protein functions as a two domain hinge in response to maltose coordination, changing from an open to a closed form (Figure 12) [92]. This action is propagated through the β-lactamase structure, restoring catalytic activity at the active site. When expressed in *E. coli*, this results in maltose-dependent β-lactamase activity, allowing for selection via growth on ampicillin and maltose containing media [92].

#### 5.1.2. Design and Construction: Integrated Transcription-Independent Protein-Based Biosensors

In the examples mentioned above, two different approaches were used to create each biosensor. For the calcium biosensor, significant structural information was already available to aid construction. It had previously been reported that several GFP variants had a site which could be used to create a circularly permuted construct, allowing integration of additional peptide sequences [90,93]. Additionally, the structure of the calcium bound calmodulin and M13 domains had previously been elucidated using multidimensional nuclear magnetic resonance spectroscopy [90,94]. Knowing that the calmodulin-M13 complex had a compact structure upon calcium binding it was reasoned that this change in confirmation may be able to act as a switch for GFP fluorescence [90]. Two versions of the sensor were subsequently made, one with a domain order of calmodulin, circularly permuted GFP, and M13, which was non-functional, and the second with an order of M13, circularly permuted GFP, and calmodulin, which was functional. In this method of construction, prior structural knowledge of each of the components, and the potential for synchronicity was integral. In this way, the method could be described as rational protein engineering. 

In contrast, the construction of the β-lactamase maltose biosensor relied less on prior structural knowledge and followed a more high-throughput approach [92]. During construction, members of a library of random circularly permuted β-lactamase genes were inserted randomly into a plasmid containing the maltose binding protein, and then transformed into *E. coli* [92]. This generated a diverse library of random maltose binding-β-lactamase fusion proteins. Cells containing a functional version of a sensor were then able to grow in media containing ampicillin and maltose, but not ampicillin alone [92]. The primary structural information used for this construction was the knowledge that the maltose binding protein undergoes a 20° hinge-like conformational change in response to maltose. However, specifically the protein was going to interface with the β-lactamase peptide when expressed as a fusion protein was not known a priori [92]. This method of construction relied heavily on the ability to assay a large range of genetic diversity.

#### 5.1.3. Limitations: Integrated Transcription-Independent Protein-Based Biosensors

Integrated TIPBs can generate very selective activity with large linear and dynamic ranges. However, there are some limitations to this design that prevent it from being ubiquitously applied. The first is a matter of background knowledge. Design and construction of most integrated TIPBs is done using known protein domains. This requires pre-existing domains that are known to bind to the target ligand and undergo a useful conformational change. It is also necessary to have domains with a known output that aligns with the intended assay, although this is generally less limiting. If these domains are known, then construction of the sensor requires either specific knowledge about protein structure and experience in protein engineering, or development of a domain-shuffling workflow and a method for screening generated sensors. Neither strategy is a trivial undertaking.

Perhaps the largest limitation to integrated TIPBs is that they are designed in a finite solution space. That is, the chances of success rely on very specific circumstances. There needs to be a change in conformation in the binding domain, which is capable of propagating a conformational change to the response domain, which is in turn modified to induce activity. These dynamics are highly context-dependent and there is no guarantee that such a solution exists, even with all possible permutations or arrangement combinations.

### 5.2. Semi-Modular Transcription-Independent Protein-Based Biosensors

#### 5.2.1. Function: Semi-Modular Transcription-Independent Protein-Based Biosensors

“Semi-modular” TIPB’s involve using linkers to coordinate receptor and response domain activity in response to the target ligand. Binding of the ligand to the receptor domain induces a conformational change that is propagated through the linker directly altering the response domain, generating a signal. These linkers vary in their rigidity and structure, depending on the context in which they are applied.

**Figure 13:**
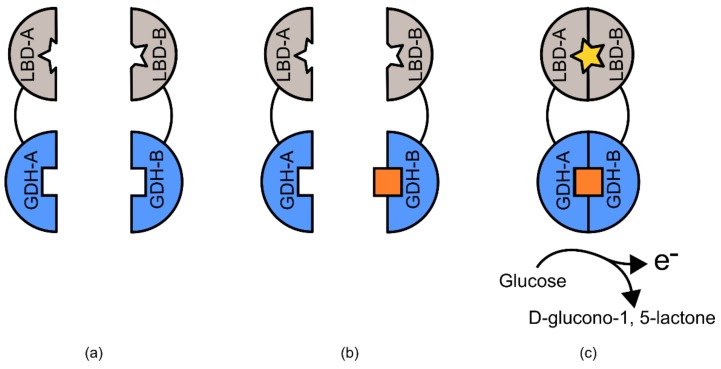
General pyrroloquinoline quinone glucose dehydrogenase (GDH)-based biosensors. Each portion of a split GDH protein is expressed with a linker to a ligand binding domain (LBD-A or LBD-B): (**a**) Under conditions of low target ligand, the GDH domains do not interact; (**b**) the addition of glucose does not restore GDH domain assembly; (**c**) target ligand (star) binding to both LBD-A and LBD-B brings GDH-A and GDH-B into close proximity, restoring electron transfer ability. Donation of an electron to an electrode acts as the signal output. Figure adapted from Guo, et al. [95].

An excellent series of examples of semi-modular TIPBs are the pyrroloquinoline quinone glucose dehydrogenase (PQQ-GDH)-based biosensors [95]. The PQQ-GDH enzyme has traditionally been used commercially in the detection of blood glucose levels, due to its ability to transfer an electron to an adjacent electrode in the presence of glucose [96,97,98]. Variants of PQQ-GDH have been used to develop biosensors for a range of target ligands. In these examples, the protein is expressed in two inactive halves, each of which is connected via linker domains, to two separate ligand-binding domains (Figure 13). Upon each domain binding the ligand of interest, the linker domains create a scaffold that brings each half of the PQQ-GDH protein into close proximity, reconstituting its ability to mediate electron transfer. This biosensing platform has been used to detect calcium, rapamycin, cyclosporine A, and FK506 [95,99].

**Figure 14:**
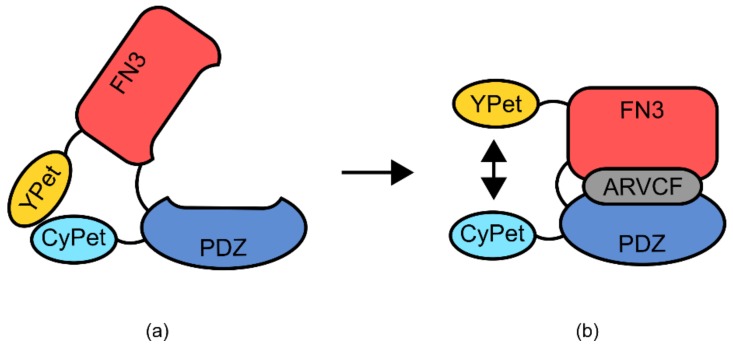
ARVCF biosensor. The biosensor utilizes the fibronectin type III domain (FN3), human erbin protein domain (PDZ), and the fluorescence resonance energy transfer (FRET) pairs YPet and CyPet. The FN3 and PDZ domains are connected by a semi rigid linker: (**a**) Under low ARVCF concentrations, this linker keeps these domains separated and brings the YPet and CyPet into close proximity; (**b**) The binding of ARVCF to the FN3 and PDZ domains provides enough stability to overcome the separating linker rigidity. This pivoting about the linker separates the YPet and CyPet domains, providing a change in FRET energy and generating a change in signal intensity. Figure adapted from Huang and Koide [100].

A second example of a semi-modular TIPBs is an affinity clamp-based biosensor for the detection of the p120-related catenin protein (ARVCF) (Figure 14) [100,101]. This sensor utilizes the PDZ domain from human erbin protein, known for its mild affinity to the ARVCF protein. Additionally, this sensor made use of a human fibronectin type III domain (FN3), which had previously been used as a generic binding scaffold for the production of similar binding proteins [100,101]. The PDZ domain was genetically fused at the N-terminus to the fluorescence resonance energy transfer (FRET) protein YPet, whilst the FN3 domain was fused to the corresponding FRET partner CyPet at the C-terminus [100]. The YPet-PDZ and FN3-CyPet proteins were joined by a small linker region. In the absence of the ARVCF protein, the PDZ and FN3 domains do not interact, but the FRET pairs CyPet and YPet come into close proximity due to heterodimerization. Upon binding of ARVCF, the protein undergoes a hinge-like conformational change, mediated via the linker region, separating the CyPet and YPet pair. This creates a usable change in FRET signal that can be assayed to monitor ARVCF concentration. 

#### 5.2.2. Design and Construction: Semi-Modular Transcription-Independent Protein-Based Biosensors

In the case of the PQQ-GDH based biosensors, the enzyme was modified in two important ways, which allowed it to be used as a generic biosensor platform. The first is the disruption of the loop present between β-strands A and B [95]. In practice this leaves the enzyme as two non-functional halves. The decision to bifurcate in this position was made after analysis of the available PQQ-GDH structure from the species *Acinetobacter calcoaceticus* [99]. The second important modification was that two complementary binding domains for a ligand of interest were linked to each half of the PQQ-GDH sequence [95]. The linkers used are of critical importance to these designs, too long, too short, too flexible, or too rigid, and the relative position of the ligand binding domains and the PQQ-GDH domains would not restore enzyme function [88].

The construction of the ARVCF biosensor incorporated a series of mutagenesis and selection screens to optimize the binding affinity of the domains used [100,101]. This was a targeted mutagenesis of several rationally chosen residues which were known to be involved in ARVCF coordination [100,101]. High functioning variants were isolated and structural analysis was performed using X-ray crystallography [100,101]. From this analysis, it became clear that the linker region used to join the YPet-PDZ and FN3-CyPet proteins was critically important for the correct function of the sensor. For a variant of the sensor, deletion of one glycine residue reduced binding affinity 8-fold, with a second deletion reducing affinity to an undetectable level [101].

#### 5.2.3. Limitations: Semi-Modular Transcription-Independent Protein-Based Biosensors

Using linkers in this manner allows for a higher degree of engineering possibilities than fully integrated designs, as both the site of integration, and linker length/composition can be modified. This means that any conformational changes that occur in the ligand binding domain have more possibilities with which to correctly modify the signal generating domains. One of the limitations of this method is that the optimal linker length and composition is difficult to know a priori, and often many different linker configurations need to be tested before an optimal sensor is generated. The linkers tested in these designs are not random sequences, but are generally informed choices based on the structural data available for both the sensing domain and response elements. This can be a challenge when utilizing known binding domains for which structural information is not available. Additionally, the design choices for linkers are non-trivial and require a moderate degree of specialized skill. This process can be assisted with accurate structural information for both the ligand binding domain and response elements, however this brings its own set of challenges when this information is unavailable.

### 5.3. Modular Transcription-Independent Protein-Based Biosensors

#### 5.3.1. Function: Modular Transcription-Independent Protein-Based Biosensors

“Modular TIPB” designs use protein tethers for receptor domain-induced co-localization of response domains, in the presence of the target ligand. As opposed to the linkers used in “semi-modular TIPB” designs, these tethers do not use conformational changes to induce response domain activity, but simply allow the recruitment of all necessary signaling components to one location. These components can be thought to be constitutively active but isolated until co-localized by the target ligand. 

**Figure 15:**
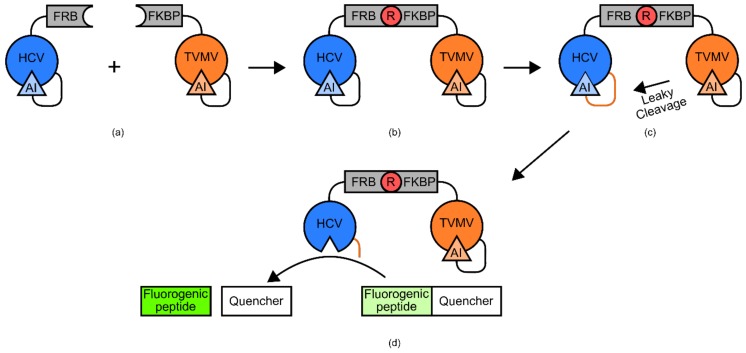
Rapamycin biosensor built using two separate fusion proteins. The first fusion protein is a rapamycin-binding FRB domain attached via a tether to a hepatitis C virus NS3 serine protease (HCV), which is in turn tethered to a HCV auto-inhibition domain (AI). The second protein is a complementary rapamycin binding FKBP12 domain, tethered to a tobacco vein mottling virus NIa protease (TVMV), which is in turn tethered to a somewhat leaky TVMV auto-inhibition domain (AI): (**a**) Under conditions of low rapamycin concentration, these proteins do not contact each other; (**b**) Under increased rapamycin concentrations, the FRB and FKBP domains simultaneously bind rapamycin, bringing the two separate fusion proteins into close proximity; (**c**) This proximity allows the leaky TVMV protease domain to cleave the tether connecting the HCV protease to its auto-inhibitor; (**d**) The loss of the auto-inhibitory domain allows the HCV protease to cleave an added fluorogenic peptide with attached quencher, restoring its fluorescence and generating a signal. Figure adapted from Stein and Alexandrov [102].

One example of such a biosensor was developed for the detection of rapamycin (Figure 15) [88,102]. The biosensor used FRB and FKBP12 domains, two previously identified complementary binding domains for rapamycin [102]. Tethered to the FKBP12 domain was a tobacco vein mottling virus NIa protease (TVMV) with a somewhat leaky auto-inhibitor domain [102]. Tethered to the FRB domain was a NS3 serine protease from hepatitis C virus (HCV), also with a tethered auto-inhibitor domain [102]. The linker peptide that tethered the HCV domain to its auto-inhibitor domain contained an amino acid sequence that was recognized by the slightly leaky TVMV auto-inhibited protease [102]. The leaky activity of auto-inhibited TVMV is not sufficient to cleave a significant number of HCV auto-inhibitor tethers [102]. However, when rapamycin is added, the two biosensor components are brought into close proximity via the stabilizing effects of the FKB12 and FRB domains [102]. This allows the slightly leaky TVMV domain to cleave the tether, securing the auto-inhibitory domain to the HCV domain [102]. HCV is then free to cleave a fluorogenic peptide with an attached quencher supplied to the solution, generating a signal [102]. 

#### 5.3.2. Design and Construction: Modular Transcription-Independent Protein-Based Biosensors

Construction of “modular TIPBs” is generally simpler than their semi-modular, or integrated counterparts. As the role of the tethers in these designs is simply to tie components together, rather than transduce conformational changes, there are fewer constraints on linker length/residue identity. Sufficiently long and generic flexible linkers are a suitable starting point for biosensor creation. It is conceivable with this type of design that a large range of generic ligand binding domains, flexible linkers, and response domains could be used in a “plug and play” fashion with little protein engineering required. Indeed, during the construction of the rapamycin biosensor mentioned above, the most complex part was the development of the competitive inhibitor for the TVMV protease domain [102]. Unlike previous biosensors described, there was comparatively very little engineering necessary to integrate the detection and response domains [102]. Furthermore, it is likely that simply substituting the FKB12 and FRB domains with a different set of complementary binding domains could produce a functional biosensor for a new target ligand. 

#### 5.3.3. Limitations: Modular Transcription-Independent Protein-Based Biosensors

The major limitations to this design strategy are baseline activation and choice of response. As the only barrier to activation is co-localization, stochastic movement of sensor components is more likely to influence biosensor output. That is, chance co-localization of sensor components will activate the sensor, resulting in signal generation. This increases the baseline noise of the sensor, decreasing the sensitivity and therefore both the linear and dynamic range. Furthermore, designs of this nature are highly dependent on known protein domains. Without efficient high-throughput methods to develop complementary binding domains, they must be sourced from nature and from corresponding literature. Alternatively, antibodies offer a possible method to generate bio-recognition elements, and have been used to generate biosensors [103]. However, this methodology also has issues with generating antibodies for small molecule ligands, as was the case with the SELEX method described above [103]. 

### 5.4. Advantages and Applicability: Transcription-Independent Protein-Based Biosensors

#### 5.4.1. Group Diagnostics: Environmental, Agricultural, and Industrial Applications

Transcription-independent protein-based biosensors are well-suited to group diagnostics from a sensory perspective. They have excellent sensitivity and specificity, with a range of signal outputs available. They have also demonstrated a wide range of possible target ligands from metals, through to larger proteins. However, the construction process may be somewhat complicated relative to the robustness necessary of the final intended assay. That is, it may be easier to design and build other types of biosensors that will be sufficient for the intended use. This is dependent, however on what literature is available regarding transcription factors, binding domains, or known aptamers.

#### 5.4.2. Point-of-Use Diagnostics: Medical, and Security Applications

Transcription-independent protein-based biosensors have shown incredible promise as point-of-use diagnostic devices. Although somewhat complex to design, when done correctly, they can possess many desirable characteristics. Some of the best examples of this are the PQQ-glucose dehydrogenase based biosensors developed by Guo, et al. [95]. Compared to their nucleic acid based biosensor counterparts, TIPBs have relatively high stability [95]. This has allowed coating and dehydration onto disposable electrodes, which can be manufactured cheaply and stored simply. These biosensors can capitalize on the already ubiquitously employed blood glucose monitoring equipment used by diabetics, allowing TIPBs designed in this way to be employed with high mobility, and with low technical expertise.

#### 5.4.3. Single-Cell Diagnostics: Metabolic Engineering, and Synthetic Biology Applications

Transcription-independent protein-based biosensors have been previously applied in single-cell diagnostic processes. Their capacity to be genetically encoded within a host cell means that they have a simple method of consistent delivery. Their rapid response times and sensitivity enables responses to slight changes within a cell. This was well demonstrated with the calcium biosensor used by Tian, et al. [6] to visualize action potentials in the neural cells of mice. The major limitation to TIPBs in single cell diagnostic applications is the lack of transcriptional output. Without transcriptional signal output it makes incorporation into genetic circuits more difficult and removes the capacity for signal generation. Additionally, for each new output that is desired, a new biosensor needs to be engineered. With the exception of a few truly modular designs, the signal input and output of TIPBs are intrinsically linked.

## 6. Conclusions

Biosensors represent both an enabling technology, and an emerging application in the field of synthetic biology. Advances in DNA synthesis, modelling of biological systems, modularity of parts, and increases in screening throughput have increased the diversity and effectiveness of biosensors. Complementary to this process has been the application of biosensors to the classic synthetic biology design-build-test cycle, dramatically increasing the throughput with which different designs can be tested. 

There is a great diversity of biosensor classes which are now applied to an equally diverse array of biotechnological applications. Most designs use DNA, RNA, protein, or transcription factors as the basis for construction. Although the limitations of different biosensor designs and their effectiveness for different applications is highly nuanced, there are some design principles that can be used to guide biosensor construction for given applications. For example, nucleic acid biosensors are readily applicable to single-cell and group diagnostics due to their simple fluorescent outputs and their ability to be genetically encoded. The SELEX process also makes nucleic acid-based biosensors the easiest to develop for target ligands, for which no known protein or transcriptional architecture is available. TIPBs are applicable to all three application-domains, but in particular to point-of-use applications due to the speed of signal actuation, low noise levels, versatility of signal outputs, and robust function, both in vivo and in vitro. However, TIPBs can be difficult to design and build, depending on the degree of protein engineering or random screening that is required to configure biosensors with desired functions. TFBs are inherently unsuitable for point-of-use diagnostics due to their slow response time and the complications that arise from using living cells. However, TFBs are exceptional at single cell diagnostics due to their natural interface with in vivo signal output (transcription), and high sensitivity and specificity to cellular concentrations of target ligands. The inherent modularity of promoters/operators and their downstream genes also makes TFBs some of the easiest biosensors to develop.

Whatever the requirement for signal detection and output, biosensors are emerging as highly sensitive, high-throughput, and cost-effective solutions to a multitude of biotechnological applications. These features are due to the inherent chemical diversity and specificity of biological molecules such as DNA, RNA, and protein, which can often outperform traditional analytics. As the tools of synthetic biology improve to further enable biosensor development, biosensors will become ever more prevalent and integral to industrial, environmental, scientific, and medical industries.
